# The spine problem: finding a function for dendritic spines

**DOI:** 10.3389/fnana.2014.00095

**Published:** 2014-09-10

**Authors:** Sarah Malanowski, Carl F. Craver

**Affiliations:** Department of Philosophy, Washington University in St. LouisSt. Louis, MO, USA

**Keywords:** dendritic spines, function, functional attribution, causal-mechanical explanation, mechanisms

## Abstract

Why do neurons have dendritic spines? This question—the heart of what Yuste calls “the spine problem”—presupposes that why-questions of this sort have scientific answers: that empirical findings can favor or count against claims about *why* neurons have spines. Here we show how such questions can receive empirical answers. We construe such why-questions as questions about how spines *make a difference to* the behavior of some mechanism that we take to be significant. Why-questions are driven fundamentally by the effort to understand how some item, such as the dendritic spine, is situated in the causal structure of the world (the *causal nexus*). They ask for a filter on that busy world that allows us to see a part’s individual contribution to a mechanism, independent of everything else going on. So understood, answers to why-questions can be assessed by testing the claims these answers make about the causal structure of a mechanism. We distinguish four ways of making a difference to a mechanism (necessary, modulatory, component, background condition), and we sketch their evidential requirements. One consequence of our analysis is that there are many spine problems and that any given spine problem might have many acceptable answers.

## Introduction

Science, according to common wisdom, does not answer why questions. Science tells us only what and how things happen. Nowhere is this common wisdom more transparently false than in anatomy and physiology, where a central pre-occupation is to understand why organisms have the parts they do. These questions have been central to research on dendritic spines from the start. Cajal’s pioneering judgment that spines are not merely artifacts of Golgi staining prompted the transparently teleological question: *why* do neurons have spines? Yuste (2011) refers to this as the “Spine Problem”: “What exactly do spines contribute to the neuron?”.

Cajal (1899) considered several answers to this question (García-López et al., 2007): that spines increase the “receptive surface” of the neuron, that they “absorb” nerve impulses, (Cajal, 1899, ibid. 119), and that they grow out to connect with distant axons. Theories of spine function have since proliferated. Shepherd, for example, reviews ten broad classes of theory, each of which can be specified in myriad ways (see Table 1, Shepherd, 1996, 2198). His list includes contributions spines might make to synaptic connectivity, development, synapse electrophysiology, synaptic plasticity, active processing of monosynaptic input, temporal processing of polysynaptic input, biochemical compartmentalization, and features of the membrane surface.

Here we explore the nature of these why-questions and the evidence required to test them. We show how these why-questions contribute to the search for neural mechanisms, distinguish several kinds of answers, and show how the mechanistic understanding of such why-questions and their answers connects those answers to empirical evidence about the causal structure of the world.

Our view of why-questions relies on the central idea that explanations in neuroscience describe mechanisms. Mechanisms consist of entities and activities organized in space and time so that they exhibit some behavior of the mechanism as a whole. Explanatory models of such mechanisms describe the spatial, temporal, and active organization of causally interacting parts. The effort to answer why-questions is a central part of the search for mechanisms that span multiple levels (Shepherd, 1983). Our focus is on these why-questions and, in particular, on what they contribute to our understanding of how the brain works, how it breaks, and how it might be improved. Why-questions, like how- and what- questions, are indispensable in the effort to understand hierarchically organized systems.

## Causal-mechanical explanation

To answer a why-question about some item (such as dendritic spines) is to represent it as part of a causal-mechanical explanation for something else. Causal-mechanical explanations in general show how a phenomenon to be explained, the *explanandum phenomenon*, is situated in the causal nexus—how it was produced, how it acts, and how it interacts with other things in the world. One can situate the explanandum phenomenon in the causal nexus in three ways: etiologically, constitutively, and contextually.

Etiological explanations show *how* the phenomenon came to pass. They look back to reveal its antecedent causes. The tipped lantern, for example, is part of the etiological explanation for the fire. Likewise, spine loss may be part of the etiological explanation for several neuropsychological disorders (Penzes et al., 2011).

Constitutive explanations answer how questions by looking down to reveal the mechanisms that underlie or maintain the phenomenon. They look inside it and describe how parts, properties, and activities are organized together in space and time such that the phenomenon occurs. Constitutive explanations often span multiple levels: higher level mechanisms are explained by lower level mechanisms, which are in turn explained by mechanisms at a lower level still, and so on. The binding of neurotransmitters to receptors on dendritic spines is part of the constitutive explanation for many instances of excitatory synaptic transmission, which are themselves involved in most of the interesting things brains do. Looking down, synaptic transmission involves numerous chemical interactions and atomic processes well beneath the current grain of explanatory interest. Multilevel mechanisms in neuroscience thus typically require research in several different fields (cellular, cognitive, etc.), since different fields are better suited for studying different levels of mechanisms.

These two aspects of causal-mechanical explanation each relate the explanandum phenomenon to the causal structure of the world in a different way. Etiological explanations look back to the causal structures that brought it about. Constitutive explanations look down to the causal structures that make it work. Each, in its own way, tells us how the explanandum phenomenon comes about.

## Why-questions and context: a third aspect of causal-mechanical explanation

The third aspect of causal-mechanical explanation is contextual. Contextual explanations describe the *role* or *function* of spines. Like constitutive explanations, contextual explanation are interlevel, but rather than looking back or looking down, contextual explanations look up and around to situate the item in question within a higher-level mechanism (Cummins, 1983; Craver, 2001). They tell us *why* neurons have spines.

Recent reviews of dendritic spine function reflect this causal-mechanical understanding of why questions and their answers. Harris and Kater (1994) argue that to understand the function of dendritic spines one must consider them, “within the context of the overall synaptic complex” including not just the spine but “the post-synaptic density, the synaptic cleft, the presynaptic axonal bouton and its vesicles, and the neighboring astrocytic complexes” (Harris 348). Each theory in Shepherd’s (1996) list situates dendritic spines within a more inclusive mechanism. Yuste (2011) focuses on one of Shepherd’s contextual mechanisms: the construction and maintenance of a distributed network with independently modifiable synapses. In each case, the goal is to look up and see how dendritic spines make a difference to the behavior of some system of antecedently acknowledged significance.

More abstractly, the answer to a why-question for an item such as a dendritic spine involves attributing a function or role to the item. This attribution can be distilled into four components:

1. A mechanism displaying a behavior in salient conditions (e.g., forming a network of modifiable synapses).
2. The mechanism’s behavior in these conditions is antecedently presumed to be important (e.g., because it affords flexible information processing).
3. Some item (part, property, or activity) is a spatio-temporal part of this system (e.g., spines, their morphology, or their growth).
4. The item makes a difference to the system behaving as it does in these conditions.

The mechanism and behavior described in (a) constitute the causal context in which the item functions. We place no constraints on the behaviors in (a) except that (b) they are antecedently presumed significant. Specifically, the behavior does not need to have contributed to the evolution (Neander, 1991) or development (Garson, 2011) of spines. Why-questions also arise in pathology and engineering: researchers study, for example, the role of spines in the etiological mechanisms of Down’s syndrome (Kaufmann and Moser, 2000) and the role spines might play in the treatment of addiction (Robinson and Kolb, 1999).

Condition (c) distinguishes contextual or functional explanation from etiological and constitutive explanation by requiring that the functional item be part of (contained within) the mechanism. Condition (d) requires that the item must make a difference to how the system behaves. If a part can make no difference at all to how the mechanism behaves, the why-question has no answer.

Constitutive, etiological, and contextual explanations are separate aspects of the same, mechanistic explanatory objective: the what, the how, and the why combine to situate an item in the causal nexus (for more recent work on evidence-based discovery see Craver and Darden, 2013; Silva et al., 2013).

## Many answers to the same why question

Why-questions ask us to situate an item within the context of a higher-level mechanism. Clearly the same item can contribute to the behavior of many mechanisms at once—it can have many *functions*. This appears to be what Shepherd (1996) has in mind when he claims that dendritic spines are *multifunctional* units. Each theory in Shepherd’s list of functions situates spines in a different causal context. This raises the reasonable prospect that there are many “spine problems” and many equally good solutions to each of them.

One reason to expect a multiplicity of functions is that distinct answers to the same why-question can be *hierarchically* related to one another. Harris and Kater (1994), for example, argue that spine shape *compartmentalizes* synaptic input, which *facilitates LTP induction* at single hippocampal synapses. Long-term potentiation, in turn, implements *weight adjustment* in computing networks that subserve *learning and memory*. These different functional attributions describe the same feature of spines in the context of different levels in one hierarchy of mechanisms (Craver, 2001, 2002; cf. Shepherd, 1983; Churchland and Sejnowski, 1992). The function attributed at one level (plasticity) requires or depends on the functions attributed in lower-level contexts (compartmentalization).

A second reason to expect that spines have multiple functions is that they make a difference to the behavior of many different systems. Spines have been hypothesized to stabilize dendrites (Kasai et al., 2010; Koleske, 2013), to protect cells from calcium toxicity (Harris and Kater, 1994; Segal, 1995), and to prevent synaptic input from short-circuiting the dendritic membrane (Yuste, 2011). These hypotheses are not competitors—spines contribute to many higher-level phenomenon, and they do so in different ways.

The fact that an item might play different roles in different higher-level containing systems has important implications for extrapolating the results of experimental findings. Given that there are many different memory systems, in different brain regions, with different underlying mechanisms, dendritic spines might play different roles in different memory systems. The same item might also play a similar functional role when described with respect to some lower-level mechanism, but an altogether different functional role when described at higher levels. Similarities and differences at multiple levels of organization can influence the extent to which experimental findings extrapolate to other systems and other organisms.

## What questions: organizational constraints on functional attribution

Many kinds of evidence can be used to argue for a particular theory concerning how an item is situated in a higher-level system. Some of this evidence is circumstantial in nature, concerning, e.g., spatial (size, shape, location, orientation) and temporal (order, rate, duration) findings about spines, their components, and their mechanistic context. Anatomical properties, such as spine locations, dimensions, morphologies, sub-structures, and organelles provide clues as to how spines might make a difference (Harris and Kater, 1994; Shepherd, 1996; García-López et al., 2007). Evidence about the time-course of intrinsic and activity-driven changes in spine morphology provides a dynamic, temporal perspective on the place of spines in the causal order of the brain (Kasai et al., 2010). Comparative evidence reveals correlations between spine density and specialization of certain brain areas (Elston et al., 2001). Such observations about *what* spines and their causal context are like can be combined with mathematical models to show how such properties would and would not influence the system’s behavior (Koch and Zador, 1993; Yuste, 2013). Findings of this sort provide global constraints on what spines can do in any context and specific constraints on what they can contribute to a particular mechanism.

Yet such findings offer only indirect tests of the causal claims at the heart of functional attributions. Correlational studies provide clues to the causal structure of a system but are almost always compatible with multiple causal structures (Eberhardt and Scheines, 2007). Models are useful for exploring a space of possibilities, but models of dendritic spine function depend crucially on idealized assumptions about the relevant structures (Harris and Kater, 1994) and on the values of unmeasured parameters (Yuste, 2013). Evidence about the time-course of spine changes, by itself, does not tell us about the causal forces that drive the development of the system over time.

## Ways of making a difference

Functional attributions—answers to why-questions—can be tested more directly by evaluating experimentally the causal claims at their heart. Ideally, these causal claims are tested by intervening to change only the putative causal variable and observing whether this change makes a difference to the effect in question. Causal experiments test directly how an item can and does make a difference within a higher-level mechanism.

Four categories of functional attribution appear to be evidentially, explanatorily, and practically distinct. On one axis, an item might make a difference either by being *necessary* for a system’s behavior or by *modulating* its behavior. Removing a necessary item prevents the behavior; removing a modulatory item merely changes it. On a second axis, an item might be either a *component* or a *background condition*. A component is a working part in the system; a background condition merely enables or assists the working parts. Combining the axes yields four kinds of functional role: necessary components, modulatory components, necessary background conditions, and modulatory background conditions.

Consider a specific example, which follows Stevens (1998) general framework. If changes in spine morphology are *necessary working components* in the mechanism of LTP, then the following causal claims should be true: (i) conditions that induce LTP should change spine morphology; (ii) preventing changes in spine morphology should prevent LTP; and (iii) inducing changes in spine morphology should be able to produce LTP in the right conditions. Condition (ii) expresses the idea of necessity in the relation; conditions (i) and (iii) combine to express the idea of componency—being a part that is sufficient in the circumstances to produce the mechanism’s behavior (Mackie, 1980).

To put this causal analysis to work, consider the hypothesis that spine enlargement is a necessary component in the mechanism of LTP induction. Spines appear to enlarge following LTP induction in accordance with condition (i) (Matsuzaki et al., 2004). And if one blocks the polymerization of actin molecules thought to be required for spine enlargement, one can prevent late LTP (Ramachandran and Frey, 2009). This provides some evidence that actin-based remodeling of spines is necessary for LTP (Bosch et al., 2014).

The status of (iii) is more complicated. We know a lot about *what* potentiated synapses are like. The volume of the spine head is directly proportional to the size of the post-synaptic density, to the number of post-synaptic receptors, to the size of the pre-synaptic bouton, to the number of vesicles generally docked at the pre-synaptic bouton, and to the quantity of neurotransmitter available for release at the synapse (Nimchinsky et al., 2002; García-López et al., 2007). However, these correlations provide only circumstantial evidence that head size makes a difference to LTP. More direct causal experiments that induce LTP by changing spine morphology are required to establish (iii) directly. It thus remains to be seen whether spine enlargement *per se* makes a difference to LTP or whether it is merely a byproduct of processes that strengthen the synapse in other ways (Redondo and Morris, 2011).

A modulatory working component satisfies (i), but not (ii) or (iii). If spine enlargement modulates LTP, then preventing spine growth would alter LTP but not prevent it (contra ii). And changes in morphology would not, by themselves, produce LTP (contra iii). However, changes in morphology should make a difference to how LTP is induced or expressed. Whether a modulatory factor is considered a background condition or a working component depends on the relationship between the factor and the other components in the system: working components work together with other components to produce the phenomenon, while background conditions are less directly implicated in the phenomenon.

A necessary background condition satisfies (ii) but not (i) or (iii). Removing a necessary background condition prevents the system from functioning. Background conditions tend to be causally independent from the operating conditions of the system: changes induced in the background conditions are therefore considered to make no difference to the system’s behavior in the relevant conditions.

This way of thinking about functional attributions and the evidence used to evaluate them can be extended to multiple levels of organization. It can be used as a framework to refine and evaluate causal evidence for distinct functional attributions. Focus on the role of dendritic spines in learning and memory. Experience-based changes in spine morphology have been proposed as a basis for long-term memory formation and retention (Bourne and Harris, 2007), but it is unclear just what this functional claim amounts to. The above framework can be used to more specifically describe how spine density changes make a difference in experience-based learning. The conditions that give rise to changes in experience also give rise to changes in spine formation (Sala and Segal, 2014), suggesting (i) is fulfilled. Other studies suggest that interfering with the formation of new spines impairs learning abilities (Soderling et al., 2007), but preventing spine formation may not eliminate learning entirely (contra ii). Studies showing that changes in spine density do not necessarily result in changes in learning and memory (Popov et al., 2007; Fester et al., 2012) suggest (iii) is not fulfilled. Spine formation thus appears to have a working modulatory role in some forms of learning and memory.

In short, this causal-mechanical analysis allows us to distinguish different kinds of functional hypotheses—different ways of making a difference to the behavior of a higher-level mechanism. Each situates an item differently within the causal context. Given that functional attributions are inherently multilevel, this analysis shows how functional attributions—answers to why-questions—integrate lower-level findings with findings about higher-level behaviors or mechanisms. Contextual explanation is inherently upward-looking and, in that sense, non-reductive.

## Conclusion

Answering why-questions, like answering how and what questions, helps to reveal the causal structure of the world. When we ask why neurons have dendritic spines, we are asking how spines fit into the workings of this most spectacular machine. Seen in this light, to claim that dendritic spines have a function in the nervous system is, implicitly, to commit one’s self to a set of causal claims about how that item makes a difference in a mechanistic context. Functional attributions gain their content from these causal claims and are evaluated most directly by testing whether those causal claims are true. Spines might play a role at many levels of organization in the nervous system and in many distinct causal systems, and might function as a necessary or modulatory component or background condition in each. Attention to the causal commitments of our functional claims helps to highlight and prioritize the diverse kinds of evidence required to evaluate them. So understood, Yuste’s spine problem is likely not one problem but many, reflecting the many ways that spines might be situated in the complex causal nexus of the brain.

## Conflict of interest statement

The authors declare that the research was conducted in the absence of any commercial or financial relationships that could be construed as a potential conflict of interest.
